# Micro-level economic factors and incentives in Children’s energy balance related behaviours - findings from the ENERGY European cross-section questionnaire survey

**DOI:** 10.1186/1479-5868-9-136

**Published:** 2012-11-21

**Authors:** Jørgen Dejgård Jensen, Elling Bere, Ilse De Bourdeaudhuij, Natasa Jan, Lea Maes, Yannis Manios, Marloes K Martens, Denes Molnar, Luis A Moreno, Amika S Singh, Saskia te Velde, Johannes Brug

**Affiliations:** 1 Institute of Food and Resource Economics, University of Copenhagen, Rolighedsvej 25, DK-1958, Frederiksberg C, Denmark; 2 University of Agder, Gimlemoen 25, Kristiansand, Norway; 3 Department of Movement and Sports Sciences, Ghent University, Watersportlaan 2, 9000, Ghent, Belgium; 4 Slovenian Heart Foundation, Dunajska 65, SI-1000, Ljubljana, Slovenia; 5 Department of Public Health, Ghent University, De Pintelaan 185, blok A, 9000, Ghent, Belgium; 6 Harokopio University 70 El, Venizelou avenue, 17671, Kallithea-Athens, Greece; 7 ResCon, Rijswijkstraat 175, 1062 EV, Amsterdam, The Netherlands; 8 Department of Pediatrics, University of Pecs, 7623 Pécs, József Attila u. 7, Pecs, Hungary; 9 Escuela Universitaria de Ciencias de la Salud, University of Zaragora, Domingo Miral s/n, 50009, Zaragoza, Spain; 10 EMGO Institute, VU University medical center, P.O. Box 7057, 1007, MB, Amsterdam, The Netherlands; 11 EMGO Institute for Care and Health Research, VUmc, P.O. Box 7057, 1007, MB, Amsterdam, The Netherlands

**Keywords:** Children, Obesogenic environment, Economic incentives, Sports activity, Soft drinks, Price responsiveness

## Abstract

**Background:**

To date, most research on obesogenic environments facing school children has focused on physical and socio-cultural environments. The role of economic factors has been investigated to a much lesser extent. Our objective was to explore the association of micro-level economic factors and incentives with sports activities and intake of soft drinks and fruit juice in 10-12 year-old school children across Europe, and to explore price sensitivity in children’s soft drink consumption and correlates of this price sensitivity.

**Methods:**

Data for the study originate from a cross-sectional survey undertaken in seven European countries (Belgium, Greece, Hungary, Netherlands, Norway, Slovenia and Spain) in 2010 among 10-12 year-old school children and their parents. In total, 7234 child questionnaires and 6002 parent questionnaires were completed. The child questionnaire included questions addressing self-reported weekly intake of soft drinks and fruit juices and time spent on sports activities, perception of parental support for sports activities, use of pocket money for soft drinks and perceived price responsiveness. Parent questionnaires included questions addressing the role of budget and price considerations in decisions regarding children’s sports activities, soft drink consumption, home practices and rules and socio-demographic background variables. Data were analysed using multiple linear regression and discrete-choice (ordered probit) modelling.

**Results:**

Economic factors were found to be associated with children’s sports participation and sugary drink consumption, explaining 27% of the variation in time for sports activities, and 27% and 12% of the variation in the children’s soft drink and juice consumption, respectively. Parents’ financial support was found to be an important correlate (Beta =0.419) of children’s sports activities. Children’s pocket money was a strong correlate (Beta =21.034) of soft drink consumption. The majority of the responding children reported to expect that significantly higher prices of soft drinks would lead them to buy less soft drinks with their own pocket money, but a majority of parents did not expect higher soft drink prices to reduce their children’s soft drink consumption.

**Conclusions:**

We conclude that economic factors, especially parents’ financial support and amount of pocket money, appear to be of importance for children’s sports participation and soft drink consumption, respectively.

## Background

Childhood overweight and obesity, caused by a positive energy balance where energy intake exceeds energy expenditure, is a concern in many countries. Recent overviews have suggested a range of specific energy balance related behaviours (EBRB) that may contribute to an increased risk for excessive weight gain [1-3], including high intake of sugared beverages, sedentary behaviour and lacking physical activity. Such behaviours have been attributed to the so-called obesogenic environment [4], where palatable high-energy foods are increasingly available, accessible and affordable relative to less energy-dense foods and beverages, while most physical activities are avoidable and no longer necessary.

A large number of studies concerning environmental correlates, determinants and interventions that may contribute to promoting healthy eating and physical activity have been reviewed in several review studies and meta-analyses. Some of these studies address the population in general, [5-8], whereas others address children and adolescents in particular [9-17], pointing at various factors of importance in the home and school environment.

The obesogenic environment has been dissected in physical, socio-cultural, political and economic environments within e.g. the ANGELO framework [4]. Most research to date has been focussed on physical and socio-cultural environments (reviewed by e.g. Ferreira et al. [10]; Huybrechts et al. [18], and Giskes et al., [19]). The reviews indicate that one of the strongest correlates of different EBRB is socio-economic position, most often represented by level of education or income. The available reviews additionally show that other, more micro-level economic factors – such as financial incentives or constraints regarding EBRB – have hardly been studied, especially where children’s EBRB is concerned, although economic factors and incentives have been suggested to be important in the context of the obesogenic environment and as tools to promote healthier eating [20,21]. A recent review specifically focussing on such incentives concluded that such economic factors may be promising in promoting healthy EBRB in children [22]. More specifically, economic motives, in terms of relative prices of energy-dense versus less energy-dense foods as well as the relation between food prices and incomes, are likely to affect the demand for foods and beverages [23]. Economic motives may also underlie the availability of foods and beverages offered to schoolchildren [24-28].

According to neoclassical micro-economic theory [23,29], rational individuals are assumed to strive at maximizing their utility (i.e. their level of needs’ satisfaction in a broad sense, including nutritional and other material needs, pleasure, convenience, etc.) within the limits given by a budgetary constraint. Within the neoclassical economic framework, we may assume that parents’ utility depends positively on their children’s current perceived utility and on the children’s future health prospects [23], and that children’s current utility, as well as their future health prospects, depend on their current consumption of sugar-sweetened drinks (soft drinks and fruit juices) and their sports activities. Within this framework, parents face a trade-off between satisfying their children’s current preferences versus their long-term health prospects, and current choices of e.g. sports activities and soft drink consumption reflect this trade-off. A number of factors may influence these trade-offs, such as budget size, with a tight budget leading to higher priority given to perceived necessities, because such necessities are perceived relatively more valuable, when the budget tightens.

The outlined micro-economic line of thinking might be considered as an element in more general social-ecological conceptual models [30]. But the economic approach could also be interpreted as a more comprehensive model on its own, with some social and cultural factors contributing to the formation of preferences, and other social factors along with availability and physical structures forming some of the framework within which individuals maximize their utility. The literature on behavioural psychology provides useful insights into some of the key mechanisms in individuals’ health behaviour that should be taken into account when extending the economic model framework in this direction, such as the Theory of Planned Behaviour [31,32] or the Health Belief Model [33], which describe health related decisions as dependent on individuals’ perception of health risk, motivation and potential barriers (including e.g. lack of self-efficacy).

If parents provide their children with money that are ‘ear-marked’ for supporting certain purposes, e.g. sports activities, the child’s perceived trade-off would tend to be more in favour of the supported activity, thus providing an economic incentive to participate in this activity.

Many parents provide their children with ‘pocket money’, which they can spend without being controlled or monitored by the parents. As children are not presumed to give high priority to their own long-term health prospects, compared with most parents’ priorities, this separation of the household budget may lead to an in-optimally high total child consumption of soft drinks, from a health promotion point of view. This occurs because the children allocate a relatively large share of their pocket money to these drinks (because they to a lesser extent prioritize future health considerations), and as parents cannot perfectly monitor these purchases, their own trade-off between children’s current preferences and long-run health does not take full account of this [34].

A change in the price relation between, for example, soft drinks versus other foods or beverages (for instance a partial increase in the price of these soft drinks) implies that the rational individual will re-allocate money from soft drink consumption to other goods. If the household budget is split into children’s ‘pocket money’ and ‘rest’, the child will respond to a price change within the trade-off between alternative pocket money spending opportunities, whereas parents respond within the framework of a reduced real household budget, taking (imprecisely) into account that the children cover some soft drink consumption with their own money.

A tighter children’s budget – in terms of fewer pocket money – may tend to make children’s own soft drink purchases more price sensitive on the one hand, because the money scarcity will increase the perceived value of this money for other purposes (opportunity cost). On the other hand, less pocket money may imply a low initial consumption and hence less potential for changes in consumption implying a lower price sensitivity. Similarly, if children spend a large share of their pocket money on soft drinks, this may imply relatively lower price responsiveness on these drinks due to lower perceived opportunity costs, but on the other hand, the high consumption implies a relatively larger room for change, leaving the net effect on price responsiveness an empirical question. Similar mechanisms related to changes in budget apply to the price sensitivity in parents’ soft drink demand, but as soft drinks normally constitute a relatively small share of parents’ household budget, the opportunity cost effect of a soft drink price change is considered to be moderate. The higher priority that parents give to their children’s currently perceived utility, compared with long-run health prospects, the less responsive will the parents’ purchases be to a soft drink price increase. Hence, in a setting where children get their own pocket money, the main effect of a price increase on soft drinks is expected to occur in the children’s own purchases.

The objective of the present paper is to explore the association between economic factors and incentives and different EBRB (sports activity and intake of soft drinks and fruit juice) in 10-12 year-old school children in seven European countries: Belgium, Greece, Hungary, Netherlands, Norway, Slovenia and Spain. Furthermore, we aimed to investigate the extent of price sensitivity in children’s soft drink consumption and the role of economic factors in determining this price responsiveness.

The above neoclassical economic framework suggests that the economic environment may influence children’s EBRB via two mechanisms: either via the relative prices/costs of alternative choices, or via the size of the budget at the decision maker’s disposal. Parents are generally presumed to make decisions that can be influenced by economic incentive mechanisms, but in many cases, parents delegate parts of these decisions to the children, either by offering earmarked funds for specific purposes, e.g. sports, or by providing pocket money for the children to spend on minor items at their own choice. Such delegation of decisions introduces two levels of decision making: the parent level and the child level. Based on this theoretical framework, we derived the following research hypotheses regarding children’s sports activities and soft drink consumption:

1. Children’s sports activity is negatively associated with a tight household budget, because such sports activities may not be perceived as a necessity.

2. Children’s time spent on sport activities is positively associated with parents’ subsidization of these activities - subsidization makes sports participation less costly from the child’s perspective.

3. Children’s consumption of soft drinks and fruit juice is negatively associated with a perceived tight household budget - because such drinks may not be considered as necessities.

4. Children’s consumption of soft drinks and fruit juice is positively associated with allocating pocket money to the children - because the children’s trade-offs tend to favourize short-term pleasure and consumption.

5. Children’s price responsiveness regarding soft drink purchases is correlated with the amount of pocket money – but the direction of this correlation depends on the balance between ‘opportunity cost effect’ and ‘room for change effect’.

6. The share of the children’s pocket money that is currently spent on soft drinks is correlated with demand’s price responsiveness - if marginal utility is assumed to be a decreasing function of the quantity of soft drink consumed (and similarly for other pocket money spendings) the substitution effect will tend to be stronger, if soft drinks constitute a large share of the children’s pocket money.

Hypotheses 1-4 primarily address the role of budgetary restraints - from the household and from the child perspective - on children’s EBRB, as measured by sports activity and consumption of soft drinks and fruit juices. Hypotheses 5-6 relate to the role of price incentives, using the stated price responsiveness of soft drink consumption as a marker, because soft drinks tend to be one of the commodities that many children most often buy with their own pocket money. These research hypotheses will be investigated in the empirical work below.

## Data and methods

### Sampling and participants

Data for the study originate from the cross-sectional survey of the ENERGY study undertaken in seven European countries (Belgium, Greece, Hungary, Netherlands, Norway, Slovenia and Spain) in March-July 2010 [35]. In the survey, child questionnaires were distributed to more than 1000 10-12 year-old school children recruited from 15-60 schools in each of the seven countries, upon informed consent of the parents and approval from ethical committees, where necessary. A parent questionnaire was sent to the parents of these children. Response rates (as a fraction of individuals approached) varied from 33% to 98% among children, and from 41% to 86% among parents [36]. For the seven countries in total, 7234 child questionnaires and 6002 parent questionnaires were completed. The background and theoretical framework, as well as the methods of the cross-sectional survey are described in more detail elsewhere [35,37,38].

The first results of the ENERGY cross sectional study regarding country differences in overweight, obesity and energy balance related behaviours have also been published elsewhere [36]. These results showed large differences in overweight/obesity and in risk behaviours between the different countries with, in general, Northern European countries showing more favourable results.

### Outcome measures

Among other issues, the questionnaires addressed children’s behaviour regarding their sports activities, their consumption of soft drinks and fruit juices, and whether a significant price increase would affect their propensity to buy soft drinks with their own money. In particular, the children were asked, whether a doubled price of soft drinks would lead them to buy less soft drinks with their own money. The choice of a 100% price increase was made, because this can be considered as a significant price increase that is also relatively easy to understand intuitively for the children. In addition, the parent questionnaire included the question, whether doubled soft drink price would lead their child to consume less soft drinks.

### Environmental correlate measures

The questionnaire also addressed a number of environmental factors and possible moderators and mediators for children’s energy-balance related behaviour, including a number of economic factors, which can be related to the respective hypotheses. Among economic variables, children were asked, to which extent they spend ‘pocket money’ on soft drinks and juice. Parents were asked, how much money they give their child to spend on foods and drinks, whether they pay for children’s sports activities - and whether cost considerations restrict their child’s sports participation or the foods given to the child. Parents were also asked about other factors that might influence their home practices regarding sports activities and food choice, including health considerations, saving time for homework, etc. In light of this, we interpret the replies to these questions as an indicator of, how binding the budget is perceived to be for the parents, relative to the other considerations that might restrict sports activity or food choice. A number of additional variables were also included in order to control for potential confounding, reflecting some of the factors suggested by socio-ecological approaches [30], such as parenting practices and socio-economic differences, as well as perceived health risk factors [39], such as child weight.

Test-retest reliability and construct validity of the questions was examined for the child questionnaire [38] and for the parent questionnaire [40]. The reader is referred to these publications for a description of the examination methodology. Test-retest reliability of the applied child questionnaire items was concluded to be “moderate” to “excellent”, except for the question, whether parents allow the child to take part in physical activity/sports, and construct validity was found to be “moderate” for most variables [38]. The applied variables from the parent questionnaire were all found to exhibit good test-retest reliability and construct validity [40].

An overview of the definitions of variables used in the subsequent analysis is given in table 1.

**Table 1:** Variable definitions

	**Question/statement**	**Answers**
**Child questionnaire**		
*Outcome variables*		
Child’s sports activity		Hours/week*
Child’s soft drink consumption		Ml/week**
Child’s juice consumption		Ml/week**
If the price of fizzy drinks and fruit squash were doubled, I would buy less fizzy drinks or fruit squash from my own money		Fully disagree/Disagree a bit/Neither agree nor disagree/Agree a bit/Fully agree
*Economic variables*		
How often do you spend your own money on fizzy drinks or fruit squash?		Never/Not often/Sometimes/Often/Always
*Socio*-*cultural variables*		
Do your parents/care givers allow you to take part in physical activity/do sports		Yes/No
If you indicate that you like a certain physical activity/sport, will your parents/care givers allow?		Never/Not often/Sometimes/Often/Always
My parents/care givers help me if I need something for my sports		Fully disagree/Disagree a bit/Neither agree nor disagree/Agree a bit/Fully agree
Do you think you are too thin or too fat		Much too thin/Bit too thin/Neither too thin nor too fat/Bit too fat/Much too fat
**Parent questionnaire**		
*Outcome variables*		
If the price of soft drinks were doubled, my child would drink less soft drinks		Fully disagree/Disagree a bit/Neither agree nor disagree/Agree a bit/Fully agree
*Economic variables*		
I let my child participate in sports less than I would like, because it is too expensive		Fully disagree/Disagree a bit/Neither agree nor disagree/Agree a bit/Fully agree
On average how much money do you give to your child to buy foods and drinks per week?		<5€/5-10€/11-20€/21-30€/31-40€/41-50€/>50€
I don’t give my child some foods because they cost too much		Fully disagree/Disagree a bit/Neither agree nor disagree/Agree a bit/Fully agree
I pay for my child to take part in sports		Never/Not often/Sometimes/Often/Always
bring my child to PA/sports sessions		Never/Not often/Sometimes/Often/Always
*Home phyisical environment variables*		
Home availability, PC-factor		Continuous (range -1;1) (cf. table 2)
*Socio*-*cultural variables*		
Parents’ soft drink consumption		ml/week**
Parents’ soft drink consumption freq.		Times/week
Parents’ juice consumption		ml/week**
Parents’ juice consumption frequency		Times/week
I give soft drink/juice to my child as a reward or to comfort him/her		Never/Not often/Sometimes/Often/Always
Home health arguments, PC-factor		Continuous (range -1;1) (cf. table 2)
Home enforcement, PC-factor		Continuous (range -1;1) (cf. table 2)
Home awareness, PC-factor		Continuous (range -1;1) (cf. table 2)
What do you think about your child’s weight?		Way too little/Bit too little/OK/Bit too much/Way too much
I would consider my child as being price conscious regarding food, snacks etc.		Fully disagree/Disagree a bit/Neither agree nor disagree/Agree a bit/Fully agree
*Socio*-*economic variables*		
Mother’s education		<7 yrs/7-9 yrs/10-11 yrs/12-13 yrs/14+ yrs
Father’s education		<7 yrs/7-9 yrs/10-11 yrs/12-13 yrs/14+ yrs
Mother’s occupation		Empl. publ sector/empl. priv. sector/self-employed/no paid job
Father’s occupation		Empl. publ sector/empl. priv. sector/self-employed/no paid job
Single parent		Yes/No
Were the biological parents born in…		Yes (2 or 1 parent)/No
Member state		BE/GR/HU/NL/NO/SI/ES

*Number of hours per week for favourite + second favourite sports.

**Times of drinking per week, multiplied by average number of ml per day of drinking.

### Statistical methods

The data were analysed statistically in three steps in order to address the 6 research hypotheses. In order to avoid possible problems with collinearity between 12 parent questionnaire variables reflecting home soft drink environment (including whether there are soft drinks available to the child at home, whether the child gets soft drinks when asked for, whether the child is allowed to take soft drinks whenever he/she wants, etc., cf. table 2), a principal components analysis was undertaken as a first step in the statistical procedure. This analysis showed that four principal components accounted for 65% of the total variation in these variables (table 2). Rotating these dimensions using varimax rotation, these four principal components can be interpreted as home availability for the child, parents’ use of health arguments to reduce child’s consumption, parents’ difficulty in enforcing agreements and their awareness of the children’s consumption, and these four orthogonal factors replaced the underlying 12 variables in the subsequent statistical analysis.

**Table 2:** Principal component analysis of home environment factors

	**Home availability**	**Home health arguments**	**Home enforcement**	**Home awareness**	**Communality estimates**
Eigen value	3.614	2.187	1.081	0.903	
% variation	30.1%	18.2%	9.0%	7.5%	
cumulative %	30.1%	48.3%	57.3%	64.9%	
Varimax rotated factor loadings					
There are soft drinks available at home for my child *	0.795	-0.188	0.025	0.092	0.68
I pay atttention to the amount of soft drinks that my child drinks*	-0.248	0.173	-0.196	0.746	0.69
If my child asks for soft drinks; I will give it to him/her*	0.815	-0.106	0.087	-0.192	0.72
My child is allowed to take soft drinks whenever (s)he wants*	0.726	-0.046	0.147	-0.380	0.70
I negotiate with my child how much soft drinks (s)he is allowed to drink*	0.060	0.258	0.326	0.680	0.64
How often do you tell your child that soft drinks are not good for him/her*	-0.191	0.758	0.104	0.278	0.70
How often do you tell your child that soft drinks can make him/her fat*	-0.079	0.819	0.090	0.070	0.69
How often do you tell your child that soft drinks are bad for his/her teeth*	-0.067	0.833	0.016	0.069	0.70
If I would like to drink soft drinks, I would restrain myself because of the presence of my child*	-0.333	0.452	0.183	0.223	0.40
If I prohibit my child from drinking soft drinks, (she) tries to drink it anyway*	0.059	0.113	0.823	0.029	0.70
If I prohibit my child from drinking soft drinks, I find it difficult to stock to my rul(s), if (s)he starts negotiating*	0.104	0.081	0.825	-0.002	0.70
How often do you or your spouse drink soft drinks with your child**	0.690	-0.091	0.038	0.017	0.49

*Response levels: Never/Not Often/Sometimes/Often/Always/Missing.

** Response levels: Never/Less than once a week/Once a week/2-4 days a week/5-6 days a week/Every day/More than once every day/Missing.

In the second step of the statistical procedure, we investigated hypotheses 1-4 by conducting multiple linear regression analyses with self-reported engagement in sport activities and soft drink and fruit juice consumption as dependent variables and variables representing children’s pocket money and parents’ payment of sports and perception of budgetary restrictions as main correlates, and variables representing physical and socio-cultural home environment to control for potential confounding.

In these statistical models, qualitative multi-level variables (e.g. ‘fully disagree’, ‘disagree a bit’, etc.) are represented by a sequence of binary “dummy” variables, representing the different response levels but one (reference response level - e.g. ‘neither agree nor disagree’). This representation of the multilevel independent variables minimizes the sensitivity of the results to the specific coding of these variables (like e.g. a linear relationship between levels). As a consequence of this representation, econometric analysis yields a beta coefficient estimate for each of these response levels.

The multiple linear regression model to address hypotheses 1-2 regarding *Child*’*s sports activity* included two economic variables, given by the responses to the statements *I pay for my child to take part in sports*, and *I let my child participate in sports less than I would like*, *because it is too expensive*. Non-economic physical and socio-cultural home environment variables, as well as attitudinal and socio-economic correlates were included in order to control for parents’ moral and practical support for the child’s sports activity. Hypothesis 1 implies significantly lower beta-coefficients for ‘agreement’ than for ‘disagreement’ levels on the dummy variables representing the statement whether parents restrict their children’s sports participation because of costs, and the hypothesis can be evaluated by the pattern of these coefficients. Hypothesis 2 implies a positive beta-coefficient on the dummy variable, representing whether parents pay for the child’s sport in this regression equation.

Regarding research hypotheses 3-4, the multiple regression model for *Children*’*s soft drink consumption* included three economic factors, represented by the responses to the statements *How often do you* (*child*) *spend your own money on fizzy drinks or fruit squash*, *On average how much money do you* (*parent*) *give to your child to buy foods and drinks per week*, and *I* (*parent*) *don*’*t give my child some foods because they cost too much*. Physical and socio-cultural home soft drink environment variables, as well as attitudinal and socio-economic correlates, were included in order to control for confounding effects, in particular of the home environment. Hypothesis 3 implies lower beta-coefficients for ‘agreement’ than for ‘disagreement’ levels with the statement that *I don*’*t give my child some foods because they cost too much*, and hypothesis 4 implies a positive beta-coefficient on the variable representing the amount of money given to the child per week for buying foods and drinks, and higher beta-coefficients for “often” and “always” levels than for “never” and “not often” levels of the *How often do you spend your own money on fizzy drinks or fruit squash* statement. The model for *Child*’*s juice consumption* contained the same variables as the soft drink model, except that the home environment was represented by parents’ juice consumption instead of the corresponding soft drink variables.

Research hypotheses 5 and 6 regarding price responsiveness were analysed in the third step on the basis of the children’s agreement with the statement *If the price of soft drinks were doubled*, *I would buy less soft drinks from my own money*, and the parents’ agreement with the statement *If the price of soft drinks were doubled*, *my child would drink less soft drinks*. The two questions address two aspects of the research hypothesis - on the one hand, whether the children will change their pattern of pocket money spending, and on the other hand whether their total consumption of soft drinks would change, i.e. if changes in the children’s purchases would be passed through to their total consumption, or whether the children will compensate for reduction in their own purchases with soft drinks available at home. As the answers to these two questions took the form of discrete interval scales (fully disagree, disagree a bit, etc.), these answers were analysed as categorical variables using an ordered probit model approach [41]. According to this approach, we assume that individual *i*‘s perceived degree of price responsiveness can be represented by the variable *y*_i_^*^, which is assumed to depend on a number of independent variables *x*_*j*_,(e.g. amount of pocket money, socio-economic correlates represented by dummy variables, cf. above, etc.) in a linear manner.

$$y_{i}^{*}=\alpha +\sum_{j}\beta_{j}\cdot x_{\mathrm{ji}}+\varepsilon_{i}$$

The *y*_i_^*^ - variable *per se* is an unobservable (latent) variable, but it is reflected in the children’s and parents’ replies to the statements *If the price of soft drinks were doubled*…, represented by the observed response variable *у*_*i*_, which takes the categorical values “fully disagree”, “disagree a bit”, etc.

*β*-parameters thus represent the impact of a particular independent variable on the probability of responding at a higher level of agreement with the statement of price responsiveness. The ordered probit method offers one way to estimate the *β*-parameters of this functional relationship within a maximum likelihood framework. Hypothesis 5 can then be evaluated by the sign and significance of the beta-coefficients associated with the *On average how much money do you give to your child to buy foods and drinks per week* variable in two statistical models, one for the child’s reply to the question, and one for the parent's answer. Hypothesis 6 can be evaluated on the basis of the beta-coefficients associated with the different response levels of the *How often do you spend your own money on fizzy drinks or fruit squash* variable. Furthermore, the beta coefficients associated with parents’ replies to the statement *I don*’*t give my child some foods because they cost too much* reflect the role of the overall household food budget on the responsiveness to price changes. In the model for parents’ answer, parents’ impression of their children’s general price consciousness was included, in order to capture possible confounding effects of child’s own responses to a price change.

It should be noted that the respondents’ categorization of price responsiveness is based on their own subjective perception of, what e.g. “agree a bit” means. So the statistical analysis of these answers primarily address e.g. the (ordinal) extent to which the respondents subjectively see themselves (or their children) as responsive to price changes on soft drinks, rather than to provide “objective” (cardinal) estimates of the price responsiveness.

The statistical analysis was done using SAS (R) statistical software.

## Results

### Soft drink consumption and sports activity

Results regarding economic variables from the multiple regression analyses are presented in table 3 (a complete list of estimated coefficients is given in Annex A). Collinearity was checked using condition index, but no serious signs of collinearity were detected.

**Table 3:** Associations (Beta coefficients and P-values) of selected economic variables with children’s engagement in sports activities, and with consumption of soft drinks and fruit juice

	**Sports activity (hours per week)**		**Soft drink consumption (ml per week)**		**Fruit juice consumption (ml per week)**	
	**Beta-coeff.**	**P-value**	**Beta-coeff.**	**P-value**	**Beta-coeff.**	**P-value**
***Economic variables***						
On average how much money do you give to your child to buy foods and drinks per week?			21.034	0.004	10.573	0.062
I pay for my child to take part in sports	0.419	<0.001				
I let my child participate in sports less than I would like, because it is too expensive						
- *I fully disagree*	0.604	<0.001				
- *I disagree a bit*	0.444	0.001				
- *I agree a bit*	0.104	0.474				
- *I fully agree*	0.289	0.084				
How often do you spend your own money on fizzy drinks or fruit squash?						
- *Never*			-1480.0	<0.001	-486.7	<0.001
- *Not often*			-986.5	<0.001	-280.2	0.021
- *Often*			1303.4	<0.001	48.1	0.835
- *Always*			648.3	0.234	-85.4	0.840
I don’t give my child some foods because they cost too much						
- *I fully disagree*			29.971	0.833	-53.432	0.636
- *I disagree a bit*			-28.820	0.860	-132.386	0.312
- *I agree a bit*			-147.352	0.388	-6.694	0.961
- *I fully agree*			238.461	0.216	-33.215	0.829
R^2^	0.19		0.26		0.09	
Economic variables’ share of total explained variation	0.28		0.28		0.12	

Hypothesis 1 states that children’s sports activity is negatively associated with a tight household budget. The pattern of the beta-coefficients related to the different levels of agreement with the *I let my child participate in sports less than I would like*, *because it is too expensive* statement, with higher – and more significant – coefficients for “I fully disagree” and “I disagree a bit” than for the other response levels yields support for this hypothesis.

Hypothesis 2, that children’s time spent on sports activities is positively associated with parents’ subsidization, is supported by the significantly positive beta-coefficient associated with the *I pay for my child to take part in sports* variable.

The third hypothesis states that children’s consumption of soft drinks and fruit juice is negatively related to a tight household budget. The non-significance of the estimated beta-coefficients – as well as the unclear pattern - associated with the different response levels of agreement with the *I don*’*t give my child some foods because they cost too much* statement, both for soft drinks and fruit juice, indicates that this hypothesis could not be supported by the statistical analysis.

Hypothesis 4, stating that children’s consumption of soft drinks and fruit juice is positively associated with the amount of pocket money, was examined by the pattern of the beta-coefficients associated with different response levels to the *How often do you spend your own money on fizzy drinks or fruit squash* statement as well as by the sign of the beta-coefficient related to the *On average how much money do you give to your child to buy foods and drinks per week* variable. In the soft drink equation, beta-coefficients associated with “Never” and “Not often” levels of the *How often do you spend your own money on fizzy drinks or fruit squash* variable were significantly negative, whereas coefficients associated with “Often” and “Always” were significantly positive and insignificant, respectively, indicating that children rarely spending their own money on soft drinks have a lower intake than children that more frequently use their own money for such drinks. A corresponding – but weaker – pattern was found in the juice consumption equation. In both the soft drink and the juice equation, the beta-coefficients associated with the variable *On average how much money do you give to your child to buy foods and drinks per week* were significantly positive (although the significance in the juice equation was relatively low), suggesting a positive association with the amount of pocket money given to the child. These findings all yield support for hypothesis 4.

The variables presented in table 3 represents economic factors in the linear regression analyses, which as mentioned constitute a subset of the entire set of explanatory variables (see Annex A [ Additional file 1] for the complete list of explanatory variables). An analysis of variance shows that in the sports activity equation, these economic factors represent 27 per cent of the total explained variation, and in the juice consumption equation, they represent 12 per cent. In the equation for soft drinks, the subset of economic factors represent 27 per cent of the total explained variation, and alone the child’s frequency of spending pocket money for soft drinks explains 25 per cent of the variation between children.

### Price responsiveness in soft drink consumption

Looking at price responsiveness in soft drink consumption as an indication of the potential effectiveness of economic incentives, the responses to questions about the children’s and parents’ expected effects of doubling the price on soft drinks are tabulated in table 4.

**Table 4:** Children’s and parents’ agreement with statement on price responsiveness of child’s soft drink consumption

	**Child:**	**Parent:**
	**If the price of soft drinks were doubled, I would buy less soft drinks from my own money**	**If the price of soft drinks were doubled, my child would drink less soft drinks**
I fully disagree	7%	33%
I disagree a bit	6%	12%
Neither agree nor disagree	17%	25%
I agree a bit	20%	17%
I fully agree	50%	14%
Total responses	100%	100%

About 70 per cent of the children agreed that a doubling of the price would make them buy less soft drinks and half of the children even “fully agree”. Parents appear however to be less convinced that higher prices will make their children consume less soft drinks, in that only about 30 per cent agree (fully or “a bit”) that a doubled price would reduce the intake. Although these answers may seem to be conflicting at a first glance, it should be kept in mind that the parents’ replies refer to children’s total intake, including soft drinks paid by e.g. the parents, whereas the children’s replies refer to the children’s own purchases.

Results of the probit analysis related to economic correlates are presented in table 5 (the full set of estimation results are given in Annex B [ Additional file 2]). Coefficients in the table represent the statistical relationship between the considered variable or response and the respondents’ subjectively and qualitatively perceived propensity to express a higher degree of agreement with the statement that a doubling of the soft drink price will reduce the child’s soft drink consumption.

**Table 5:** Potential effect of economic variables on expected price responsiveness in children’s soft drink consumption

	**Child:**		**Parent:**	
	**If the price of soft drinks were doubled, I would buy less soft drinks from my own money**		**If the price of soft drinks were doubled, my child would drink less soft drinks**	
	**Beta-coeff.**	**P-value**	**Beta-coeff.**	**P-value**
**Economic variables**				
On average how much money do you give to your child to buy foods and drinks per week?	-0.0006	0.442	-0.0025	0.189
How often do you spend your own money on fizzy drinks or fruit squash?				
**-***Never*	-0.2528	<0.001	-0.0829	0.076
**-***Not often*	-0.1473	0.009	-0.1150	0.027
**-***Often*	0.1658	0.074	-0.1249	0.136
**-***Always*	0.0643	0.388	-0.2626	0.098
I don’t give my child some foods because they cost too much				
**-***I fully disagree*	-0.0293	0.344	0.5317	<0.001
**-***I disagree a bit*	-0.1580	0.030	0.2226	<0.001
**-***I agree a bit*	-0.0397	0.325	-0.1877	0.002
**-***I fully agree*	-0.0905	0.181	-0.4132	<0.001
I would consider my child as being price conscious regarding food, snacks, etc.				
**-***I fully disagree*			0.1512	0.026
**-***I disagree a bit*			0.0329	0.325
**-***I agree a bit*			-0.0721	0.085
**-***I fully agree*			-0.0999	0.033
Log-likelihood	-3008.920		-5308.922	

As regards hypothesis 5, that increased pocket money is related to children’s price responsiveness in soft drink consumption, the coefficient of -0.0006 associated with the variable *On average how much money do you give to your child to buy foods and drinks per week* variable in the child response equation suggests a negative (albeit not significant) effect of the amount of money on the child’s propensity to agree more with the statement, and hence on the child’s expected price responsiveness. A similar finding was obtained in the equation representing parents’ response to the question, whether increased price would be associated with lower soft drink intake of the child. Hence, these statistical results do not yield support for hypothesis 5.

According to the results in table 5, the pattern of beta-coefficients associated with the variable *How often do you spend your own money on fizzy drinks or fruit squash* in the child response equation shows a significantly negative effect of the “Never” and the “Not often” response levels, whereas responses at the remaining levels did not show significant influence. A similar pattern was obtained in the parent response equation. This suggests that children, who rarely or never buy soft drinks with their own money, are less price responsive than children more frequently buying soft drinks using their own money, thus yielding some support for hypothesis 6.

## Discussion

Based on economic theory, this study derives and explores six hypotheses regarding the influence of economic factors on children’s EBRB, as expressed by their level of sports activity and consumption of soft drinks and fruit juices. Four of these hypotheses addressed the role of budgetary conditions on the children’s EBRB, and the statistical analysis yields support for three of these hypotheses: that the sports participation of some children is negatively related to parents’ cost considerations, that the sports participation is positively associated with parents’ paying, and that the consumption of soft drinks and juice is positively related to the amount of pocket money. The statistical analysis did not yield support for the fourth hypothesis, that children’s consumption of soft drinks or juice is related to the tightness of the household budget.

The study’s finding that economic constraints can be a barrier for children’s sports activities is in line with some recent findings, e.g. that lack of economic and material resources in the family contributed significantly to explaining social stratification in sports participation [42]. Out of a large number of studies reviewed by Ferreira and co-authors, some also suggested a relationship between socio-economic status and physical activity (which is of course broader than sports), but a considerable share did not find significant evidence for a positive association between socio-economic status and physical activity in children and adolescents [10].

The lack of support for hypothesis 3 may be somewhat surprising from an economic-theoretical perspective. However, the results may be considered as fairly consistent with findings in a number of studies reviewed by Giskes and co-authors [5], which did not find a significant relationship between socio-economic status and diet quality in terms of energy and fat intake. This may either suggest that this consumption cannot be explained by economic theory, or that the soft drink consumption yields the children a relatively low-cost short-tem utility gain, compared with other food products, and hence is relatively little sensitive to the size of the food budget.

To the authors’ knowledge, there exists very little empirical evidence on the role of pocket money as a determinant for children’s soft drink consumption. But the results in this study suggest a significant role of such pocket money.

Two research hypotheses dealt with the role of economic factors for the price responsiveness of soft drink consumption. 70% of the children and 30% of the parents think that significant price increases on soft drinks would lead the children to reduce their soft drink purchases and intakes, respectively. A hypothesis that the price responsiveness of soft drinks is related to the share of children’s pocket money spent on soft drinks was supported by the statistical analysis. Children, who often use their own money for soft drinks, tend to be more price responsive than children who less often buy soft drinks with their own money and this suggests that “room for change” is more important than “opportunity cost” in the children’s response to price changes. The statistical analysis however did not yield support for the hypothesis that price responsiveness was associated with the total amount of pocket money given to the child.

Regarding parents’ responses, whether their child would consume less soft drinks if the price were higher, parents who consider their budget tight with respect to food costs tend to expect lower price responsiveness than parents who find the household budget less tight. One possible interpretation of this finding could be that families with relatively restricted food budgets exercise more firm home rules on the consumption of e.g. soft drinks, irrespective of price level. A number of further explanatory variables were included in the statistical analysis of perceived price responsiveness. For example, parents from a relatively ‘soft drink tolerant’ home environment significantly seemed to expect lower price responsiveness in their children’s soft drink consumption.

The above results suggest that micro-level child and parent-reported economic factors are significantly and rather substantially associated with children’s EBRB, such as soft drink consumption and sports activities. Some of these findings are generally in accordance with *a priori* expectations derived from economic theory whereas others represent new insights. Hence, according to these results and if confirmed in more robust longitudinal or experimental research, such economic factors should be taken into consideration, when formulating new initiatives to promote healthier EBRB among children across Europe. Especially the role of children’s pocket money regarding soft drink intake and parents’ economic and practical support for their children’s sports activities appear to be relevant.

The statistical analysis in this paper was based on a cross-section dataset, which implies that we can only analyse correlations, but it is not possible to identify causal relationships between the variables. It should also be noted that many of the analysed replies are based on respondents’ self-reports and thus reflect subjective categorizations and expectations of the response possibilities, for example in relation to the price responsiveness questions. This may have biased the results. Nevertheless, we believe that the present study’s attempt to include economic data in a large-scale multi-country observational study is a useful first step in exploring the possible importance of such economic factors as drivers of children’s energy balance related behaviours. Future research in this area might attempt to further quantify the degree of price responsiveness, either by stating more explicit and quantitative choice options in upcoming questionnaire surveys, or possibly applying a continuous variable approach in order to determine price elasticities.

## Conclusions

Based on data from the cross-sectional survey among seven European countries, we conclude that micro-level economic factors tend to be associated with children’s physical activity and consumption of soft drinks and fruit juice. Children’s pocket money may constitute an important driver of soft drink consumption and parents’ financial support may be an important determinant for children’s sports activities. The majority of the responding children expect that significantly higher prices of soft drinks will lead them to buy less of these soft drinks with their own pocket money, and this effect seems to be stronger for ‘heavy user’ children, who buy soft drinks relatively often. In contrast, the majority of parents do not think that higher prices of soft drinks will lead their children to consume less soft drinks and parents who consider their household budget as binding tend to expect a smaller effect of price increases than parents, who do not restrict food purchases due to cost considerations.

## Competing interests

The authors declare that they have no competing interests.

## Authors’ contributions

JDJ conducted the statistical analysis and drafted the manuscript. StV, ASS, IDB, MKM, YM, EB, LM, LAM, NJ and DM were responsible for data collection in the seven countries and assisted in providing the final version of the manuscript. JB coordinated the project and assisted in editing the manuscript. All authors read and approved the final manuscript.

## Supplementary Material

Additional file 1 **Annex A.** Detailed linear regression results. Click here for file

Additional file 2 **Annex B.** Detailed ordered probit model regression results. Click here for file
