# Supplementary material for: Micro-level economic factors and incentives in Children’s energy balance related behaviours - findings from the ENERGY European cross-section questionnaire survey
Source: Int J Behav Nutr Phys Act. 2012 Nov 21;9:136. doi: 10.1186/1479-5868-9-136 (PMC3514146; doi:10.1186/1479-5868-9-136)
Supplement: Additional file 1 — Annex A. Detailed linear regression results. [file 1479-5868-9-136-S1.docx]

| Annex A. Detailed linear regression results | |  | |  | |  |  |  |  |
| --- | --- | --- | --- | --- | --- | --- | --- | --- | --- |
|  | Sports activity (hours per week) | | | | Soft drink consumption (ml per week) | | | Fruit juice consumption (ml per week) | |
|  | Beta-coeff | | P-value | | Beta-coeff | | P-value | Beta-coeff | P-value |
| Intercept | -0,373 | | 0,416 | | 3202,262 | | <0.001 | 1818,558 | <0.001 |
| On average how much money do you give to your child to buy foods and drinks per week? |  | |  | | 21,034 | | 0,004 | 10,573 | 0,062 |
| I pay for my child to take part in sports | 0,419 | | <0.001 | |  | |  |  |  |
| I let my child participate in sports less than I would like, because it is too expensive |  | |  | |  | |  |  |  |
| *- I fully disagree* | 0,604 | | <0.001 | |  | |  |  |  |
| *- I disagree a bit* | 0,444 | | 0,001 | |  | |  |  |  |
| *- I agree a bit* | 0,104 | | 0,474 | |  | |  |  |  |
| *- I fully agree* | 0,289 | | 0,084 | |  | |  |  |  |
| How often do you spend your own money on fizzy drinks or fruit squash? |  | |  | |  | |  |  |  |
| *- Never* |  | |  | | -1479,952 | | <0.001 | -486,743 | <0.001 |
| *- Not often* |  | |  | | -986,538 | | <0.001 | -280,220 | 0,021 |
| *- Often* |  | |  | | 1303,447 | | <0.001 | 48,129 | 0,835 |
| *- Always* |  | |  | | 648,265 | | 0,234 | -85,358 | 0,840 |
| I don't give my child some foods because they cost too much |  | |  | |  | |  |  |  |
| *- I fully disagree* |  | |  | | 29,971 | | 0,833 | -53,432 | 0,636 |
| *- I disagree a bit* |  | |  | | -28,820 | | 0,860 | -132,386 | 0,312 |
| *- I agree a bit* |  | |  | | -147,352 | | 0,388 | -6,694 | 0,961 |
| *- I fully agree* |  | |  | | 238,461 | | 0,216 | -33,215 | 0,829 |
| Do your parents/care givers allow you to take part in physical activity/do sports | 0,015 | | 0,959 | |  | |  |  |  |
| My parents/care givers help me if I need something for my sports | 0,316 | | <0.001 | |  | |  |  |  |
| If you indicate that you like a certain physical activity/sport, will your parents/care givers allow? | 0,267 | | <0.001 | |  | |  |  |  |
| I bring my child to PA/sports sessions | 0,175 | | <0.001 | |  | |  |  |  |
| Home availability, PC-factor |  | |  | | 473,738 | | <0.001 |  |  |
| Home health arguments, PC-factor |  | |  | | -31,544 | | 0,547 |  |  |
| Home enforcement, PC-factor |  | |  | | 199,617 | | <0.001 |  |  |
| Home awareness, PC-factor |  | |  | | -119,149 | | 0,015 |  |  |
| I give soft drink/juice to my child as a reward or to comfort him/her |  | |  | |  | |  |  |  |
| *- Never* |  | |  | | -101,474 | | 0,468 | -86,563 | 0,433 |
| *- Not often* |  | |  | | -102,218 | | 0,608 | 71,225 | 0,625 |
| *- Often* |  | |  | | 700,095 | | 0,166 | 466,944 | 0,088 |
| *- Always* |  | |  | | 40,043 | | 0,956 | -630,396 | 0,143 |
| Parents’ soft drink/juice consumption frequency |  | |  | | 92,725 | | 0,005 | 99,178 | <0.001 |
| Parents' soft drink/juice consumption |  | |  | | 0,060 | | 0,043 | 0,092 | <0.001 |
| Do you think you are too thin or too fat |  | |  | |  | |  |  |  |
| *- Much too thin* | 0,280 | | 0,099 | | 291,534 | | 0,156 | -129,140 | 0,431 |
| *- Bit too thin* | 0,258 | | 0,027 | | 55,036 | | 0,698 | -41,987 | 0,714 |
| *- Bit too fat* | -0,286 | | 0,006 | | -170,940 | | 0,180 | 20,541 | 0,842 |
| *- Much too fat* | -0,242 | | 0,246 | | 634,936 | | 0,014 | -181,094 | 0,377 |
| What do you think about your child’s weight? |  | |  | |  | |  |  |  |
| *- Way too little* | -0,053 | | 0,828 | | 169,518 | | 0,565 | -43,582 | 0,848 |
| *- Bit too little* | -0,202 | | 0,410 | | 165,237 | | 0,574 | -248,622 | 0,272 |
| *- Bit to much* | -0,189 | | 0,492 | | 429,377 | | 0,201 | 67,919 | 0,794 |
| *- Way too much* | -1,055 | | 0,019 | | 727,551 | | 0,169 | -573,176 | 0,184 |
| Mother's education |  | |  | |  | |  |  |  |
| *- less than 7 years* | -0,470 | | 0,229 | | -585,657 | | 0,204 | -599,553 | 0,090 |
| *- 7-9 years* | -0,176 | | 0,398 | | 353,953 | | 0,150 | 93,482 | 0,621 |
| *- 10-11 years* | -0,319 | | 0,030 | | 105,070 | | 0,559 | -11,723 | 0,934 |
| *- 12-13 years* | -0,258 | | 0,012 | | -29,959 | | 0,813 | 43,197 | 0,670 |
| Father's education |  | |  | |  | |  |  |  |
| *- less than 7 years* | -0,146 | | 0,691 | | 170,396 | | 0,675 | -38,045 | 0,907 |
| *- 7-9 years* | 0,150 | | 0,460 | | 781,772 | | <0.001 | 9,949 | 0,957 |
| *- 10-11 years* | -0,119 | | 0,352 | | 270,672 | | 0,084 | 128,769 | 0,304 |
| *- 12-13 years* | 0,157 | | 0,120 | | 56,056 | | 0,653 | 269,206 | 0,007 |
| Mother's occupation |  | |  | |  | |  |  |  |
| *- Empl public sector* | -0,013 | | 0,918 | | -53,745 | | 0,718 | 47,430 | 0,688 |
| *- Empl private sector* | 0,009 | | 0,938 | | -80,444 | | 0,574 | 224,733 | 0,048 |
| *- Self-employed* | 0,035 | | 0,817 | | 127,739 | | 0,488 | 256,190 | 0,081 |
| Father's occupation |  | |  | |  | |  |  |  |
| *- Empl public sector* | 0,208 | | 0,337 | | -186,969 | | 0,506 | -229,459 | 0,288 |
| *- Empl private sector* | 0,190 | | 0,366 | | -27,385 | | 0,920 | -212,635 | 0,311 |
| *- Self-employed* | 0,310 | | 0,155 | | -335,084 | | 0,235 | -133,813 | 0,537 |
| Single parent | -0,085 | | 0,697 | | -235,677 | | 0,402 | 60,331 | 0,786 |
| NotNative | -0,191 | | 0,194 | | 265,846 | | 0,124 | 243,856 | 0,076 |
| Greece | -0,350 | | 0,021 | | -1821,348 | | <0.001 | -129,183 | 0,393 |
| Hungary | 1,365 | | <0.001 | | 879,916 | | <0.001 | 726,597 | <0.001 |
| Netherlands | 0,020 | | 0,909 | | 1228,736 | | <0.001 | 578,868 | 0,002 |
| Norway | 1,095 | | <0.001 | | -958,578 | | <0.001 | -488,095 | 0,002 |
| Slovenia | 1,322 | | <0.001 | | -586,793 | | 0,003 | 715,463 | <0.001 |
| Spain | 0,021 | | 0,881 | | -1136,675 | | <0.001 | -30,089 | 0,841 |
