# Supplementary material for: Micro-level economic factors and incentives in Children’s energy balance related behaviours - findings from the ENERGY European cross-section questionnaire survey
Source: Int J Behav Nutr Phys Act. 2012 Nov 21;9:136. doi: 10.1186/1479-5868-9-136 (PMC3514146; doi:10.1186/1479-5868-9-136)
Supplement: Additional file 2 — Annex B. Detailed ordered probit model regression results. [file 1479-5868-9-136-S2.docx]

| Annex B. Detailed ordered probit model regression results | | |  |  |  |
| --- | --- | --- | --- | --- | --- |
|  | **Child** | | | **Parent** | |
|  | If the price of soft drinks were doubled, I would buy less soft drinks with my own money | | | If the price of soft drinks were doubled, my child would drink less soft drinks | |
|  | Beta-coef | P-value | | Beta-coef | P-value |
| Intercept | -1,3285 | <0.001 | | -0,1567 | 0,200 |
| Intercept2 | 0,3202 | <0.001 | | 0,3417 | <0.001 |
| Intercept3 | 0,9389 | <0.001 | | 1,1027 | <0.001 |
| Intercept4 | 1,4824 | <0.001 | | 1,7387 | <0.001 |
| On average how much money do you give to your child to buy foods and drinks per week? | -0,0006 | 0,442 | | -0,0025 | 0,189 |
| How often do you spend your own money on fizzy drinks or fruit squash? | | | | |  |
| *- Never* | -0,2528 | <0.001 | | -0,0829 | 0,076 |
| *- Not often* | -0,1473 | 0,009 | | -0,1150 | 0,027 |
| *- Often* | 0,1658 | 0,074 | | -0,1249 | 0,136 |
| *- Always* | 0,0643 | 0,388 | | -0,2626 | 0,098 |
| I don't give my child some foods because they cost too much | |  | |  |  |
| *- I fully disagree* | -0,0293 | 0,344 | | 0,5317 | <0.001 |
| *- I disagree a bit* | -0,1580 | 0,030 | | 0,2226 | <0.001 |
| *- I agree a bit* | -0,0397 | 0,325 | | -0,1877 | 0,002 |
| *- I fully agree* | -0,0905 | 0,181 | | -0,4132 | <0.001 |
| I would consider my child as being price conscious regarding food, snacks, etc. | | | | |  |
| *- I fully disagree* |  |  | | 0,1512 | 0,026 |
| *- I disagree a bit* |  |  | | 0,0329 | 0,325 |
| *- I agree a bit* |  |  | | -0,0721 | 0,085 |
| *- I fully agree* |  |  | | -0,0999 | 0,033 |
| Home availability, PC-factor | -0,0106 | 0,361 | | -0,1633 | <0.001 |
| Home health arguments, PC-factor | -0,0583 | 0,015 | | -0,0788 | <0.001 |
| Home enforcement, PC-factor | -0,0352 | 0,086 | | -0,1893 | <0.001 |
| Home awareness, PC-factor | -0,0458 | 0,035 | | 0,0454 | 0,009 |
| I give soft drink/juice to my child as a reward or to comfort him/her | |  | |  |  |
| *- Never* | -0,0485 | 0,248 | | -0,1277 | 0,009 |
| *- Not often* | -0,0177 | 0,434 | | -0,2486 | <0.001 |
| *- Often* | -0,2553 | 0,151 | | -0,3565 | 0,036 |
| *- Always* | -0,2509 | 0,218 | | 0,0301 | 0,457 |
| Parents’ soft drink/juice consumption frequency | 0,0087 | 0,307 | | -0,0132 | 0,153 |
| Parents' soft drink/juice consumption | 0,0000 | 0,133 | | 0,0000 | 0,296 |
| Do you think you are too thin or too fat |  |  | |  |  |
| *- Much too thin* | 0,0239 | 0,406 | | -0,0702 | 0,193 |
| *- Bit too thin* | -0,0763 | 0,140 | | 0,0120 | 0,414 |
| *- Bit too fat* | -0,0932 | 0,080 | | 0,0320 | 0,262 |
| *- Much too fat* | -0,2601 | 0,034 | | 0,0303 | 0,383 |
| What do you think about your child’s weight? |  |  | |  |  |
| *- Way too little* | 0,1064 | 0,241 | | -0,2066 | 0,036 |
| *- Bit too little* | -0,0199 | 0,448 | | -0,1384 | 0,113 |
| *- Bit to much* | 0,0386 | 0,411 | | -0,2717 | 0,019 |
| *- Way too much* | 0,2817 | 0,152 | | 0,0214 | 0,460 |
| *Mother's education* |  |  | |  |  |
| *- less than 7 years* | 0,214 | 0,171 | | -0,3992 | 0,013 |
| *- 7-9 years* | 0,042 | 0,358 | | -0,1957 | 0,022 |
| *- 10-11 years* | 0,094 | 0,144 | | -0,1078 | 0,061 |
| *- 12-13 years* | 0,038 | 0,278 | | -0,0499 | 0,157 |
| Father's education |  |  | |  |  |
| *- less than 7 years* | 0,140 | 0,240 | | 0,2354 | 0,070 |
| *- 7-9 years* | 0,155 | 0,082 | | -0,0334 | 0,359 |
| *- 10-11 years* | 0,125 | 0,055 | | -0,0453 | 0,228 |
| *- 12-13 years* | 0,071 | 0,136 | | 0,0543 | 0,132 |
| Mother's occupation |  |  | |  |  |
| *- Empl public sector* | -0,202 | 0,004 | | 0,0136 | 0,408 |
| *- Empl private sector* | -0,165 | 0,011 | | -0,0315 | 0,286 |
| *- Self-employed* | -0,042 | 0,328 | | 0,0339 | 0,319 |
| Father's occupation |  |  | |  |  |
| *- Empl public sector* | -0,063 | 0,322 | | 0,0148 | 0,446 |
| *- Empl private sector* | -0,083 | 0,265 | | 0,0187 | 0,430 |
| *- Self-employed* | -0,051 | 0,354 | | -0,0238 | 0,414 |
| Single parent | 0,196 | 0,080 | | -0,0538 | 0,313 |
| NotNative | 0,118 | 0,082 | | -0,2937 | <0.001 |
| Greece | 0,356 | <0.001 | | -0,0165 | 0,416 |
| Hungary | 0,058 | 0,300 | | -0,2575 | <0.001 |
| Netherlands | -0,018 | 0,448 | | 0,1593 | 0,040 |
| Norway | 0,100 | 0,191 | | -0,2060 | 0,004 |
| Slovenia | -0,006 | 0,478 | | -0,0560 | 0,233 |
| Spain | 0,191 | 0,044 | | 0,0080 | 0,458 |
